# Genome Characterization of Enterococcus faecalis Bacteriophage EFKL

**DOI:** 10.1128/mra.00160-23

**Published:** 2023-05-03

**Authors:** Loh Michelle JiaMin, Prasanna Mutusamy, Priyadarshini Karthikeyan, Ramesh Kumaresan, Andrew Millard, Sivachandran Parimannan, Heera Rajandas

**Affiliations:** a Centre of Excellence for Omics-Driven Computational Biodiscovery, AIMST University, Kedah, Malaysia; b Faculty of Dentistry, AIMST University, Kedah, Malaysia; c Department of Genetics and Genome Biology, University of Leicester, Leicester, United Kingdom; d Center for Evolutionary Hologenomics, Globe Institute, University of Copenhagen, Copenhagen, Denmark; DOE Joint Genome Institute

## Abstract

We characterized the complete genome of the lytic Enterococcus faecalis phage EFKL, which was isolated from a sewage treatment plant in Kuala Lumpur, Malaysia. The phage, which was classified in the genus *Saphexavirus*, has a 58,343-bp double-stranded DNA genome containing 97 protein-encoding genes and shares 80.60% nucleotide similarity with *Enterococcus* phage EF653P5 and *Enterococcus* phage EF653P3.

## ANNOUNCEMENT

Bacteria of the genus *Enterococcus* colonize the human gastrointestinal tract, oral cavity, and genitourinary tract (1). Enterococcus faecalis and Enterococcus faecium are the most common species that cause numerous life-threatening infections, with E. faecalis being predominant (2). The resistance of E. faecalis to common antibiotics and increased antibiotic resistance in biofilms have further complicated the treatment of these infections (3, 4). In view of phage therapy being one of the promising alternatives to combat antibiotic-resistant biofilm-associated infections, we isolated the phage EFKL from a crude sewage water sample collected on 31 August 2021 at a sewage treatment plant located in Kuala Lumpur, Malaysia (3.1842°N, 101.7090°E).

Briefly, the sample was enriched with the host E. faecalis ATCC 29212 in brain heart infusion (BHI) broth (Oxoid, UK) at 37°C for 24 h and centrifuged at 8,000 rpm at 4°C for 10 min. The supernatant was filtered (0.2-μm-pore-size filter) and subjected to plaque assay (5). The plaque morphology was clear and circular, with a mean diameter of 1 mm (6). High-titer phage stock was prepared by eluting the phage in saline magnesium (SM) buffer from a double-layer agar plate with confluent lysis (7). Genomic DNA of phage EFKL was extracted from the high-titer phage stock using phenol-chloroform (8), and the DNA concentration was measured with a Qubit 4 fluorometer. DNA libraries were prepared using the NEBNext Ultra II DNA library preparation kit, and sequencing was performed on an Illumina NovaSeq 6000 platform with paired-end 2 × 150-bp reads. The quality of the raw reads was assessed using FastQC v0.11.9 (9) before trimming using BBDuk from the BBMap v39.01 package (ktrim=r hdist=1 tpe tbo minlen=100 qtrim=rl trimq=28) (https://github.com/BioInfoTools/BBMap/blob/master/docs/guides/BBDukGuide.txt). The resulting reads were then subsampled to 50,000 reads from paired FASTQ files using Seqtk (https://github.com/lh3/seqtk) and were assembled into a single contig with 257.1-fold coverage using SPAdes v3.15.5 with the –only-assembler option (10), followed by polishing with Pilon v1.24 (11). CheckV v1.0.1 predicted that the assembled genome has a completeness of 100%, with a high confidence level (0 to 5% error) (12).

**FIG 1:**
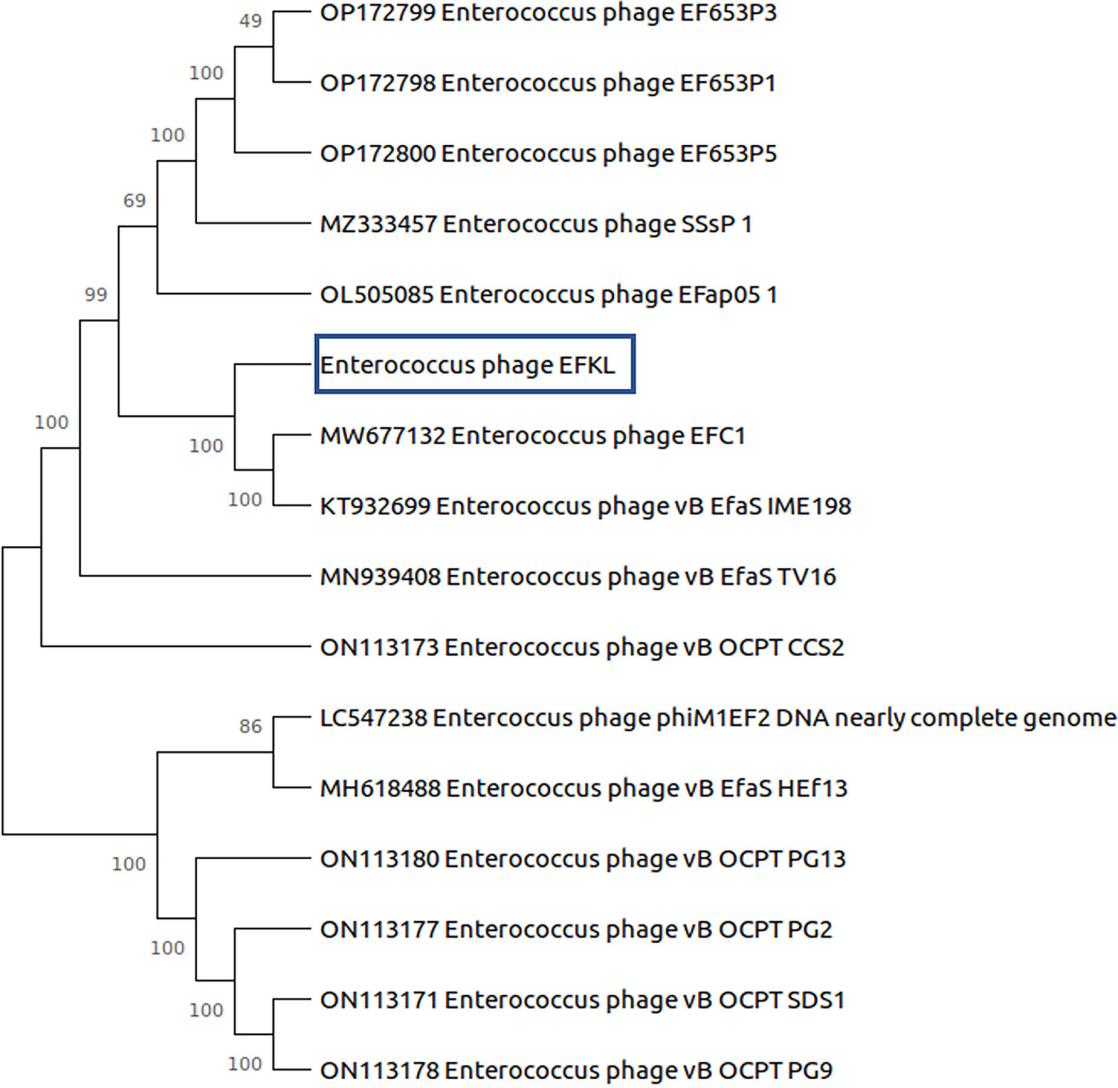
Phylogenetic analysis of *Enterococcus* phage EFKL. Core genomes were identified and aligned using Roary v3.13.0 with the –e and –n options. The core gene alignment output file from Roary was then used as the input to build the phylogenetic tree. The tree was constructed using the neighbor-joining method with 1,000 bootstrap replications and was visualized in MEGA X v10.2.6. All phages listed belonged to the genus *Saphexavirus*. The numbers next to the branches are bootstrap values.

Phage EFKL is a complete, linear, double-stranded DNA of 58,343 bp, with a GC content of 39.70%, and it contains 97 coding sequences (CDSs) and 1 tRNA gene. Genome annotation using Prokka v1.12 (13) with the PHROGs database (14) revealed that 35 CDSs were annotated as known functional genes, while the remaining CDSs encode hypothetical proteins. Additionally, no lysogenic lifestyle, antimicrobial resistance, or virulence genes were detected in the genome when it was analyzed with PhageLeads (15). BLASTn (https://blast.ncbi.nlm.nih.gov/Blast.cgi) search of the complete genome against the nonredundant NCBI database (accessed 20 January 2023) revealed that EFKL was most closely related to *Enterococcus* phage EF653P5 (GenBank accession number OP172800) and *Enterococcus* phage EF653P3 (GenBank accession number OP172799), with 80.6% nucleotide identity, as computed by the VIRIDIC web service (accessed 20 January 2023) (16). A phylogenetic analysis was performed by aligning 23 core genes of 15 closely related enterococcal phages using Roary v3.13.0 (17). A phylogenetic tree was generated and visualized using MEGA X v10.2.6 (18). *Enterococcus* phage EFC1 and *Enterococcus* phage vB_EfaS_IME198 were found to be the closest relatives of phage EFKL (Fig. 1). All of these related enterococcal phages belong to the genus *Saphexavirus*; therefore, phage EFKL is classified as a virus from the genus *Saphexavirus*.

### Data availability.

The complete genome of phage EFKL has been deposited in the GenBank database under the accession number OP831581. The associated BioProject, SRA and BioSample accession numbers are PRJNA934410, SRP422348, and SAMN33275621, respectively.
